# Burden of depression and anxiety among leprosy affected and associated factors—A cross sectional study from India

**DOI:** 10.1371/journal.pntd.0009030

**Published:** 2021-01-22

**Authors:** Karthikeyan Govindasamy, Immanuel Jacob, Raju Moturu Solomon, Joydeepa Darlong

**Affiliations:** 1 Research Domain, The Leprosy Mission Trust India, New Delhi, India; 2 Counselling department, TLM Community Hospital, Vadathorasalur, Tamil Nadu, India; Hospital Infantil de Mexico Federico Gomez, MEXICO

## Abstract

**Background:**

Leprosy is a Neglected Tropical Diseases (NTDs) known to cause stigma and discrimination in low-and middle-income countries. It often results in visible impairments, thus pre-disposing to poor mental health. Aim of the study was to estimate the prevalence of depression and anxiety among people affected by Leprosy and to determine the associated factors.

**Methodology/Principal findings:**

A multi-centric, cross-sectional study was carried out in four leprosy endemic states of India—Chhattisgarh, Maharashtra, West Bengal and Tamil Nadu in randomly selected blocks (a sub-unit of district), from one district in each state. From selected blocks those registered for leprosy treatment at public health or referral centres, people above the age of 18 years were interviewed with PHQ-9 and GAD-7 questionnaires for Depression and Anxiety, respectively. Disease profile like leprosy classification, deformity grade, number and site of the patches and socio-economic status were collected along with individual data.

Of the total 220 respondents, prevalence of depression and anxiety symptoms was, 33% (73) and 19% (42), respectively. Presence of disability (47%) and Female gender (46%) were significantly associated with depression. Presence of disability (32%), Lower income group (27%) and low education (22%) were significantly associated with symptoms of anxiety. As the severity of disability increased, risk of developing depression and anxiety increased.

**Conclusion:**

The study indicates that more than 30% of people affected by leprosy have mental health problems, which emphasizes the importance of mental health care services in leprosy. Women, those who had lower level of education, those belonging to lower socio-economic status and those with any level of disability due to leprosy are at risk of developing depression and/or anxiety. The study concludes more attention to be paid to the categories identified to be at risk.

## Author summary

Mental health of people affected by leprosy has not been studied much. There is no prevalence data, and the psychological burden of leprosy is unknown, along with factors contributing to it. Estimating and understanding this data is essential to plan interventions and resources to address it. This study was conducted to obtain the prevalence of depression and anxiety in four leprosy endemic states of India. Patient Health Questionnaire-9 and General Anxiety Disorder-7 was used to screen for depression and anxiety. Study participants were leprosy affected persons who had completed their course of antibiotics from the government and tertiary care hospitals from the study areas. A total of 220 patients were interviewed out of which 33% and 19% were found to be suffering from depression and anxiety, respectively. The factors that contributed to depression and anxiety were women, those with disability and poverty. A person having depression was more prone to anxiety. This study highlights an important issue of the neglected areas of healthcare that need urgent attention. Addressing the mental health of persons affected by leprosy will help in holistic care and wellbeing of people.

## Introduction

Leprosy is one of the oldest and most neglected diseases known to cause stigma and discrimination, especially in Low-and Middle income countries as compared to other stigmatizing diseases[1,2]. The resulting visible impairments and the stigma associated with the disease are root cause of psychosocial problems such as exclusion from the family, community and work[3,4]. WHO in its current Global leprosy program recognized stopping discrimination and promotion of inclusion as a core component in its program[5]. Leprosy and other Neglected Tropical diseases which have lifelong stigmatizing sequalae, have been associated with the poor mental health[6]. The visible impairments, stigma and discrimination pre-disposes to poor mental health among people affected by leprosy[7]. WHO in its recent report highlighted the mental health issues and its impact on persons affected by leprosy, including suicidal tendency[8]. In developing countries where mental health issues are less talked about and in itself associated with stigma[9], the affected person faces a dual problem of dealing with the leprosy and mental health problems such as depression and anxiety. On the other hand, among the general population, mental illness is among the leading causes of ill health and disability worldwide, affecting more than 450 million people across the globe[10]. In low resource countries, where leprosy is more prevalent, there are few and sometimes even absence of mental health services due to limited political and financial investment and a lack of qualified personnel. Adequate evidence is lacking to this effect globally on the prevalence of mental health disorders and the treatment needs of persons living with leprosy to effectively intervene to minimize its impact.

The prevalence of mental ill health has been reported by many authors in India. Soni et al, in a study on migrated leprosy patients reported that 14% of the subjects had moderate to severe depression and almost 1 in 5 patients who showed symptoms of depression had attempted suicide[11]. Levy EL et al reported association between neuropathic pain and psychological morbidity such as anxiety and depression was detected in 15% of patients and 41% of patients with neuropathic pain had psychological morbidity[12]. Studies from other developing countries where leprosy is prevalent like Brazil, Bangladesh and Ethiopia also reported prevalence of psychiatric morbidity[13–15]. These studies suggest a need for better mental health care of the leprosy afflicted. Due to lack of understanding and good data about mental health needs of the leprosy afflicted, such issues have not been adequately addressed. The reported prevalence of mental health problems among the persons affected by leprosy are mostly hospital or leprosy colony based studies, which cannot be generalized. Planning of any intervention programme and necessary justification needs representative data which can serve as baseline. In order to get an overall estimate in the country it is essential to collect the same from states with different cultures, in vast countries like India. The leading research question of this study is “What is the prevalence of Mental health issues in people living with leprosy and its correlates?” The objective of the study is to estimate the prevalence of common mental health problem such as depression and anxiety in different regions and associated factors among people affected by leprosy.

## Methods

### Ethics statement

The study was approved by The Leprosy Mission Trust India Ethics committee, reference number; 5/vi/C-35. Informed oral consent was obtained from all the study participants recruited into the study. All data used were anonymised.

A multi-centric, cross sectional study was carried out in 4 states of India endemic for leprosy viz. West Bengal (WB), Chhattisgarh (CG), Tamil Nadu (TN) and Maharashtra (MH), with the annual new case detection rate of 11.38, 43.69, 6.27 and 12.22 per 100,000 population, respectively, during the fiscal year 2016–17. In each state, one district where the Leprosy Mission Hospital is situated was selected from which one block (sub-unit of district) was randomly selected, which has an average population of over 100,000 population. All the patients from the selected block registered for treatment at public health system and/or hospitals of The Leprosy Mission Trust India during the period of January 2016 to December 2017 were line listed, sequenced based on date of registration. Among those, above 18 years of age, who completed full course of multi drug therapy were traced and interviewed after obtaining the consent. The data collection was done between April to July 2019. When patient was not traceable, the next patient in line was included in the study. The sample size was powered (80%) to assess the prevalence of mental health impairment of 20%, with an absolute precision of 5% on either side and an alpha level of 0.05%. Recruitment was done till the desired sample size of 55 participants in each study site was achieved.

### Data collection

The data collection tools includes; 1) Individual data sheet containing details of the individual, data collected through review of medical charts related to disease profile like leprosy category, detection delay (duration between first symptom to diagnosis), presence of ulcer (wound) and site of the patches were recorded from the medical charts available and verified with the patients during the interview. The disability was assessed at the time of interview and graded as per standard WHO classification of disability in leprosy as grade 0,1 and 2. For hand and foot: Grade 0—No anaesthesia, no visible deformity or damage, Grade 1 –Anaesthesia, but no visible deformity or damage and Grade 2 –Visible deformity or damage present. For eye: Grade 0 –No eye problems due to leprosy; no evidence of visual loss, Grade 1 –Eye problems due to leprosy present, but vision is not severely affected as a result (vision 6/60 or better) and Grade 2 –Severe visual impairment (vision worse than 6/60). The disability grade is assigned separately for each eye, hand and foot. The highest grade assigned is taken as disability grading for the patient. In the EHF (sum) score, we add grades assigned for all six parts together, making a possible score between 0 and 12. The duration of disability was recorded for all those with disability at the time of interview, defined as the time duration between impairments were first noticed and the interview. The Socio-economic status (SES), type of occupation and education status was collected during the interview. 2) Standardized Questionnaire for depression—(PHQ-9) administered to screen for symptoms of Depression. 3) Standardized questionnaire for Anxiety Disorder–(GAD-7) administered to screen for anxiety. The dataset is provided in S1 Dataset.

### Measurement of depression and anxiety

The PHQ-9 consists of 9 questions and scored as”not at all” (0), “several days” (1), “more than half the days” (2), “nearly every day (3). A total score was then generated with cut-offs classifying severity of depression. The prevalence of depression was calculated as per the guidelines of PHQ-9. A total score of 10 and above was considered depression to determine the prevalence and the factors associated[16].

The GAD-7 consists of 7 questions and scored as “Not at all sure” (0), “Several days” (1), “more than half the days” (2) and “Nearly every day” (3). A total score was generated with cut-offs suggesting different severity of anxiety. The prevalence of anxiety was calculated as per the guidelines of GAD-7. A total score of 10 and above was considered anxiety to determine the prevalence and factors associated[17].

### Statistical analysis

Descriptive statistics were used to estimate the prevalence of depression and anxiety and provided with 95% confidence interval, where required. The univariate logistic regression analysis was performed to study factors associated with the depression and anxiety. The factors (explanatory variables) were classified into meaningful groups to study its association with the mental ill health. For continuous variables which is not normally distributed, median value was used to categorize into two groups to study the association for example age. Type of house was taken as proxy for socio-economic status along with monthly income of the family. Those with concrete roof (pucca house) were considered belonging to higher SES. Standard definitions were used where available for example, type of leprosy, disability level and EHF score to classify into groups to study its association with the mental health. Those factors found to be associated with depression and anxiety in the univariate analysis were included in the multivariate linear regression (bootstrapped) model to estimate its association with scorers of PHQ-9 and GAD-7. We employed Bootstrap resampling methods[18] in linear regression model to account for skewed distribution of scores of PHQ-9 and GAD-7. The association between two continuous variables which were not normally distributed was assessed using the Spearman Rank correlation coefficient test. The p-value of <0.05 was considered statistically significant.

## Results

A total of 220 patients were included in the analysis, with 55 from each state. The demographic and clinical profile of the patients are discussed below (Table 1).

**Table 1:** Demographic and clinical profile of study participants.

Demographic and clinical characteristics		WB (n = 55)	CG (n = 55)	TN (n = 55)	MH (n = 55)	Total (n = 220)
Gender	Male	40 (73%)	31 (56%)	31 (56%)	39 (71%)	141 (64%)
	Female	15 (27%)	24 (44%)	24 (44%)	16 (29%)	79 (36%)
Age group	18 to 30 years	17 (31%)	19 (35%)	19 (34%)	17 (31%)	72 (33%)
	31 to 50 years	20 (36%)	26 (47%)	23 (42%)	26 (47%)	95 (43%)
	50+ years	18 (33%)	10 (18%)	13 (24%)	12 (22%)	53 (24%)
Education level	0	23 (42%)	15 (27%)	0	8 (15%)	46 (21%)
	1–5	12 (22%)	13 (24%)	1 (2%)	21 (38%)	47 (21%)
	6–10	17 (30%)	17 (31%)	31 (56%)	19 (34%)	84 (38%)
	11 & above	3 (6%)	10 (18%)	23 (42%)	7 (13%)	43 (20%)
Type of house	Kuccha*	39 (71%)	0	22 (40%)	41 (75%)	102 (46%)
	Pucca^	16 (29%)	55 100%)	33 (60%)	14 (25%)	118 (54%)
Occupation	Agriculture	23 (42%)	14 (26%)	21 (38%)	15 (27%)	73 (33%)
	Labourer	10 (18%)	16 (29%)	12 (22%)	27 (49%)	65 (30%)
	Housewife	15 (27%)	12 (22%)	2 (4%)	5 (9%)	34 (16%)
	Employed	0	6 (11%)	4 (7%)	1 (2%)	11 (5%)
	Self-emp./Business	2 (4%)	4 (7%)	1 (11%)	6 (11%)	13 (6%)
	Student	5(9%)	3 (6%)	14 (26%)	1 (2%)	23 (10.5%)
	Unemployed	0	0	1 (2%)	0	1 (0.5%)
Marital Status	Never married	8 (15%)	8 (15%)	15 (27%)	10 (18%)	41 (19%)
	Married	47 (86%)	44 (80%)	40 (73%)	41 (75%)	172 (78%)
	Separated/Widowed	0	3 (5%)	0	4 (7%)	7 (3%
Type of leprosy	PB	2 (4%)	16 (34%)	26 (47%)	4 (7%)	51 (23%)
	MB	53 (96%)	36 (66%)	29 (53%)	51 (93%)	169 (77%)
Detection delay	1 year & below	3 (6%)	12 (22%)	26 (47%)	36 (65%)	77 (35%)
	1 to 2 years	38 (69%)	23 (42%)	29 (53%)	14 (26%)	104 (47%)
	Above 2 years	14 (25%)	20 (36%)	0	5 (9%)	39 (18%)
WHO disability grade	Grade 0	26 (47%)	45 (82%)	39 (71%)	21 (38%)	131 (60%)
	Grade I	8 (15%)	7 (13%)	13 (24%)	10 (18%)	38 (17%)
	Grade II	21 (38%)	3 (6%)	3 (6%)	24 (44%)	51 (23%)
EHF score	0	27 (49%)	44 (80%)	39 (71%)	21 (38%)	131 (59%)
	1–2	18 (33%)	9 (16%)	12 (22%)	24 (44%)	63 (29%)
	3 & above	10 (18%)	2 (4%)	4 (7%)	10 (18%)	26 (12%)
Visible patch	Present	21 (38%)	53 (96%)	45 (82%)	49 (89%)	168 (76%)
	Absent	34 (62%)	2 (4%)	10 (18%)	6 (11%)	52 (24%)
Ulcer	History of ulcer	12 (22%)	4 (7%)	1 (2%)	20 (36%)	37 (17%)
	No ulcers	43 (78%)	51 (93%)	54 (98%)	35 (64%)	183 (83%)

^ Kuccha house–hut / thatched roof.

* Pucca house–house with concrete / tin sheeted / tiled roof.

The mean (standard deviation) age of the study participants was 39 (13) and there was no difference between the states. Out of 220 participants, 36% were females, comparatively higher in CG and TN (44% each) than WB (27%) and MH (29%). Fifty eight percent of study participants had studied above secondary education with variation between the states of TN (98%), followed by CG (49%), MH (47%) and WB (36%). About 46% were living in a Kuccha house (hut/thatched) and others in a Pucca house (tin sheeted/tiled/concrete). There was a wide variation between states in type of house.

A total of 169/220 (77%) had Multibacillary (MB) form of leprosy. Majority of participants in WB (96%) and MH (93%) were MB, whereas in CG and TN it was 66% and 53%, respectively. The overall mean (standard deviation) detection delay at the time of diagnosis (first symptom to diagnosis) was 19 (13) months, which was highest in CG 25(9), followed by WB 21(9) and MH 19(20) and least in TN 11(4). The difference in detection delay between the states were statistically significant (p- < 0.001). The proportion of people with WHO grade I or grade II disability were higher in MH (62%) and WB (53%) and least in CG and TN, 6% each. Sixty-five (29%) patients had EHF (Eye, Hand Foot) score between 1 and 2 and 26 (12%) had score of 3 or more. There was a significant association between presence of disability and study sites (p- <0.001). Among all respondents, 168/220 (76%) of patients had visible anaesthetic patch on their limb at the time of inclusion in the study and 17% (37) had history of or presence of ulcer.

### Prevalence of depression and anxiety

The level of depression among the study participants disaggregated by the different states is shown in the Table 2. Overall, 33% (95% CIs, 27% - 40%) shown signs of depression; out of which 50/220 (23%) patients showed moderate and 23/220 (10%) moderately severe to severe depression according to PHQ-9. The prevalence was highest in TN (64%), followed by MH (42%), WB (18%) and least in CG (9%). There was a statistically significant (p—<0.001) association between prevalence of depression and the states included in the study. The median (interquartile range) total PHQ-9 score was 7 (10–4).

**Table 2:** Prevalence of different levels symptoms of depression.

Depression status	Depression level (PHQ-9 score)	WB (n = 55)	CG (n = 55)	TN (n = 55)	MH (n = 55)	Total (n = 220)
Not Depression	No significant (0–4)	36 (66%)	29 (53%)	5 (9%)	6 (11%)	76 (35%)
	Mild (5–9)	9 (16%)	21 (38%)	15 (27%)	26 (47%)	71 (32%)
Depressed	Moderate (10–14)	4 (7%)	5 (9%)	22 (40%)	19 (35%)	50 (23%)
	Moderately severe to severe (15 & above)	6 (11%)	0	13 (24%)	4 (7%)	23 (10%)

The prevalence of Anxiety is shown in Table 3. Overall, 19% (95% CIs, 14% -25%) of patients shown signs of anxiety. Total of 13% (29) of patients showed moderate and 6% (13) showed severe levels of anxiety. The prevalence of moderate to severe anxiety was higher among participants from MH (40%), followed by, TN (19%) and WB (17%). The prevalence of anxiety was least among participants from CG (2%). There was a statistically significant association (p—<0.001) between prevalence of anxiety between study participants from different states. The median (interquartile range) total GAD-7 score was 5 (9–3).

**Table 3:** Prevalence of different levels symptoms of anxiety.

Anxiety level	WB (n = 55)	CG (n = 55)	TN (n = 55)	MH (n = 55)	Total (n = 220)
No significant (0–4)	35 (63%)	20 (36%)	23 (42%)	8 (14%)	86 (39%)
Mild (5–9)	11 (20%)	34 (62%)	22 (40%)	25 (46%)	92 (42%)
Moderate (10–14)	7 (13%)	1 (2%)	8 (15%)	13 (24%)	29 (13%)
Severe (15 & above)	2 (4%)	0	2 (4%)	9 (16%)	13 (6%)

### Determinants of depression and anxiety

Table 4 shows the factors associated with depression and anxiety. Patients who scored 10 and above in PHQ-9 and GAD-7 questionnaires were considered to have depression and anxiety, respectively and correlated with personal demographic, socio-economic and disease related factors. In the univariate analysis, women were found to be at 2.4 (95% CIs, 1.3–4.2) times higher risk of developing depression than men. Among the disease related factors, patients with any level of disability (grade I and II disability) had 2.9 (95% CIs, 1.6–5.1) times higher risk of developing depression than those with no disability. As EHF score (severity of disability) increased, the risk of developing depression increased. It is important to note that up to 24% of those without disability also showed symptoms of depression. Other factors such as age, socio-economic status, education level, marital status, type of leprosy, presence of visible patch, history of ulcer and detection delay were not associated with the depression.

**Table 4:** Factors associated with depression and anxiety among people affected by leprosy.

Determining Factors		Depression (n = 73)		p-value	Anxiety (n = 42)		p-value
		OR	95% CIs		OR	95% CIs	
**Gender**	Male (n = 141)	1	-	**0.004**	1	-	0.298
	Female (n = 79)	2.4	1.3–4.2		1.4	0.7–2.9	
**SE status**	Pucca house (n = 118)	1	-	0.140	1	-	**0.011**
	Kuccha house (n = 102)	1.5	0.9–2.7		2.5	1.2–4.9	
**Education levels**	11 & above (n = 43)	1	-	0.647	1	-	**0.034**
	0–10 (n = 177)	1.2	0.6–2.4		3.8	1.1–12.8	
**Type of Leprosy**	PB (n = 51)	1	-	0.515	1	-	0.135
	MB (n = 169)	1.3	0.6–2.5		2.0	0.8–5.1	
**Visible patch**	Not Present (n = 52)	1	-	0.154	1	-	0.665
	Present (n = 168)	1.7	0.8–3.4		0.8	0.4–1.8	
**Disability Grade**	Grade 0 (n = 131)	1	-	**<0.001**	1	-	**<0.001**
	Grade 1 & 2 (n = 89)	2.9	1.6–5.1		3.8	1.9–7.8	
**EHF score**	0 (n = 131)	1	-	-	1	-	-
	1–2 (n = 63)	2.5	1.3–4.7	**0.005**	2.9	1.3–6.4	**0.008**
	3 & above (n = 26)	4.5	1.9–10.8	**<0.001**	6.0	2.3–15.7	**<0.001**
**History of Ulcers**	No (n = 183)	1	-	0.625	1	-	0.977
	Yes (n = 37)	0.8	0.4–1.8		1.0	0.4–2.4	
**Detection delay**	1–12 months (n = 77)	1	-	0.103	1	-	0.059
	> 12 months (n = 143)	0.6	0.3–1.1		0.5	0.3–1.0	

Patients belonging to lower socio-economic status (OR 2.5, 95CIs, 1.2–4.9) and patients who had lower level of education (OR 3.8, 95 CIs, 1.1–12.8) were at higher risk of developing anxiety. Among the disease factors, presence of disability was a strong predictor of anxiety, at 3.8 (95% CIs, 1.9–7.8) times higher risk as compared to those without disability. As the disability (EHF score) increased the risk of developing anxiety increased. There was no significant association observed between type of leprosy, detection delay, presence of visible patch and history of ulcer. Those factors that were associated with the depression and anxiety at univariate analysis with the p-value of <0.10 were included in the multiple linear regression model using bootstrap resampling methods, adjusted for age and type of leprosy, shown in Table 5. Women, those with lower education level, those belong to poor socio-economic status, those with shorter detection delay and severity of disability (EHF score) were found to be independent factors associated with the depression (R square = 0.213). For anxiety, only increasing disability level were independently associated (R square = 0.136).

**Table 5:** Multivariate linear regression model (Bootstrapped) to assess the determinants of depression and anxiety.

Determinants	Depression		Anxiety	
	B (Beta coefficients)	p-value	B (Beta coefficients)	p-value
Constant	2.9 (-0.53–6.87)	0.128	5.72 (2.52–9.1)	0.001
Gender (women)	2.2 (0.85–3.56)	0.002	-	-
Lower Education level	0.25 (0.08–0.42)	0.002	-	-
Lower Socio-economic status	1.37 (-0.04–2.63)	0.045	-	-
Detection delay	-0.07 (-0.16 –-0.03)	0.008	-	-
EHF score	1.42 (0.84–1.95)	0.001	1.26 (0.73–1.75)	0.001

There was a statistically significant association between the presence of symptoms of depression and anxiety, r_s_ = 0.63 (p-value <0.01), indicating a strong positive correlation between ranking of PHQ-9 and GAD-7 scores.

## Discussion

The prevalence of depression and anxiety observed in this study was 33.1% and 19%, respectively. This is higher than the estimated global and Indian general population for depression between 4.4% and 4.7%, respectively. Similarly for anxiety disorders, 3.6% and 3.0% is the global and Indian population estimate[19,20]. The pattern of female to male ratio was 1.8:1 which is comparable with the other studies in the general population[19–21]. The observed level is more than the reported prevalence in Indian general population[21]. Therefore, it is reasonable to attribute the higher prevalence in this study to leprosy and its consequences. This high prevalence was also observed in studies among people affected by, other NTDs [22–24] and chronic conditions like diabetes[25]. The reasons for selecting PHQ-9 and GAD-7 is that they are standard tools for screening depression and anxiety, respectively. Also, their availability of validated version in the concerned languages of the study area, has been recommended by International Federation of Anti-Leprosy Association (ILEP) and Neglected Tropical Diseases NGO Network (NNN).

Among the demographic factors, female gender is the strong predictor of depression in leprosy. The findings are consistent with the other reports[20,26,27]. Mental ill health adds to the problems of leprosy in females as triple jeopardy[28] that they are already dealing with gender issue, disability and stigma. This has far reaching consequences and demands immediate attention and intervention. The lower socio-economic and education status appeared to be associated with anxiety disorders, as found in other population based studies[21,27]. However, their significance disappeared in multivariate analysis. In the absence of a stigma measure, the presence of disability, among the disease related factors, irrespective of its duration is the strongest factors associated with depression and anxiety. There was a positive correlation between the proportion of participants experiencing mental health problems and the severity of leprosy-related disability measured by the EHF score, thus emphasizing the importance of early detection of leprosy to prevent associated co-morbidities such as depression and anxiety. Detection delay was associated with the depression. However, the direction of association was contrary to expected. Those with shorter delay to diagnosis since noticing the first symptom in the body had higher PHQ-9 score. This may be due to the counselling / health education services provided during their visit to health facility, suggesting possible coping mechanisms that they develop as they continue living with the chronic nature of the disease or imprecision of recall of the duration. However, the coping skills was not measured and the findings were similar for those with anxiety. There was a clear association between presence of anxiety and the depression. Therefore, identification of symptoms of anxiety at an early stage could help prevent the development of depression.

The Sustainable Development Goals (SDGs) state that; By 2030, (we should) promote mental health and wellbeing for all at all ages. This is more pertinent for people living with leprosy and its sequalae as they experience stigma and discrimination along with the physical disability and at higher risk of developing mental health problems[2,3,29].They are often excluded and their basic rights violated, culminating in a struggle to overcome leprosy. For leprosy to be adequately addressed and defeated we need to identify the mental health problems of people and address them.

The study participants were relatively young (76% between18 to 50 years). The lower proportion of female patients reflects the general epidemiological features of leprosy in the study area as comparable with the national report in India, 39.17%, during the fiscal year 2016–17. The incidence of leprosy is high among younger population in endemic countries[30,31] and relatively less females among those who voluntarily report for treatment, as in the case of study participants. The proportion of MB patients and the prevalence of grade I and grade II disability are higher than the reported national report. Thus, the participants in the study sample had comparatively more severe form of disease than those of the study population. This is due to inclusion of patients from referral hospitals for leprosy from the study area where patients with advanced disease report as compared to public health system which could affect the generalisability.

Integrated care for persons with leprosy and mental health issues would be helpful as it could improve satisfaction levels of the persons affected, treatment compliance and optimal use of resources. It is prudent to identify leprosy as a medical condition with important mental health needs[32]. Studies on suicides and suicidal behaviour highlights the importance for addressing depression and intervene before it is too late[33]. Developing an integrated framework addressing leprosy and mental health issues facilitates providing holistic care for the persons affected. Leprosy treatment guidelines should include processes for screening, diagnosis and management of mental health among persons affected by leprosy. An algorithm will help health workers to screen persons affected by leprosy for mental health issues. It will be useful to collect and document more information on mental health issues related to leprosy. Counselling tailored for specific populations especially like females[34], for those with irreversible deformities and their family members would be useful for preventing and managing concurrent mental health and leprosy issues.

This report is not without limitations. The PHQ-9 and GAD is a screening and not the confirmatory tool for diagnosis of depression and anxiety, respectively, which would have over-estimated the prevalence. In addition, the study participants had an average more severe form of disease due to inclusion of more patients from referral hospital, which could have increased the prevalence of mental ill health among study participants. The demographic characteristics were comparable between the study participants from public health facility and TLM hospitals except for socio-economic status where percentage of patients from farmer facility were belong to higher socio-economic status. The clinical characteristics were different between the group; the percentage of MB cases (83.5% Vs 59.7%) and presence of disability (44.3% Vs 30.7%) and severity of disability were higher among those from TLM hospitals (shown in S1 Supplementary Tables). Accordingly, the findings in this study should be interpreted with caution and the prevalence of depression and anxiety reported in this study may not be generalized to all people affected by leprosy in India. However, the mean PHQ-9 (8.95 Vs 6.57, p-value <0.05) and GAD-7 (7.1 Vs 5.83, p-value >0.05) score was higher among those from public health facility (the results are shown in S1 Supplementary Tables). The self-esteem was not measured in this study. A higher self-esteem has been found to reduce the level of stigma and mental health problem experienced[24,29,35]. The stigma associated with leprosy and its role in mental health problem among people affected by leprosy was not studied in this investigation which is one of limitations of this study. We conducted one off interview with patient to collect data. Administering stigma scale along with PHQ-9 and GAD-7 would be difficult for our study population to process and respond to the questionnaire. Therefore, we could not measure the stigma. The study was conducted among those who had completed their Multidrug Therapy. Those who were defaulters continue to suffer from complications and sequelae could have a higher level of depression and that is a limitation of our study also. We recommend future studies using greater representative samples. Such studies should assess all factors that may contribute to poor mental wellbeing, including stigma and chronic (neuropathic) pain.

In conclusion, this study indicates the amount of burden and possible unmet needs for mental health care for people affected by leprosy. Women, those with lower level of education, those belonging to lower socio-economic status and those with any level of disability due to leprosy are at higher risk of developing symptoms of depression and/or anxiety.

## Supporting information

S1 STROBE checklist Cross-sectional design. (DOC)

S1 Dataset (XLSX)

S1 Supplementary Tables (DOCX)
