# Supplementary material for: Burden of depression and anxiety among leprosy affected and associated factors—A cross sectional study from India
Source: PLoS Negl Trop Dis. 2021 Jan 22;15(1):e0009030. doi: 10.1371/journal.pntd.0009030 (PMC7857628; doi:10.1371/journal.pntd.0009030)
Supplement: S1 Supplementary Tables — (DOCX) [file pntd.0009030.s003.docx]

**Supplementary tables**

Table 1: Difference between patients who were registered at public health facility and TLM hospitals

| **Demographic and clinical characteristics** | | **Government**  **(n=62)** | **TLM**  **(n=158)** | **Total**  **(n=220)** | **p-value** |
| --- | --- | --- | --- | --- | --- |
| Gender | Male | 40 (64.5%) | 101 (63.9%) | 141 (64%) | 0.934 |
|  | Female | 22 (35.5%) | 57 (36.1%) | 79 (36%) |  |
| Age group | 18 to 30 years | 20 (32.3%) | 52 (32.9%) | 72 (33%) | 0.304 |
|  | 31 to 50 years | 31 (50%) | 64 (40.5%) | 95 (43%) |  |
|  | 50+ years | 11 (17.7%) | 42 (26.6%) | 53 (24%) |  |
| Education level | 0 | 6 (9.7%) | 40 (25.3%) | 46 (21%) | 0.062 |
|  | 1-5 | 14 (22.6%) | 33 (20.9%) | 47 (21%) |  |
|  | 6-10 | 26 (41.9%) | 58 (36.7%) | 84 (38%) |  |
|  | 11 & above | 16 (25.8%) | 27 (17.1%) | 43 (20%) |  |
| Type of house | Kuccha* | 21 (33.9%) | 81 (51.3%) | 102 (46%) | 0.020 |
|  | Pucca^ | 41 (66.1%) | 77 (48.7%) | 118 (54%) |  |
| Occupation | Agriculture | 21 (33.9%) | 52 (32.9%) | 73 (33%) | 0.459 |
|  | Labourer | 20 (32.3%) | 45 (28.5%) | 65 (30%) |  |
|  | Housewife | 5 (8.1%) | 29 (18.4%) | 34 (16%) |  |
|  | Employed | 3 (4.8%) | 8 (5.1%) | 11 (5%) |  |
|  | Self-emp./Business | 5 (8%) | 8 (5%) | 13 (6%) |  |
|  | Student | 7 (11.3%) | 16 (10.1%) | 23 (10.5%) |  |
|  | Unemployed | 1 (1.6%) | 0 (%) | 1 (0.5%) |  |
| Marital Status | Never married | 12 (19.4%) | 29 (18.4%) | 41 (19%) | 0.660 |
|  | Married | 47 (75.8%) | 125 (79.1%) | 172 (78%) |  |
|  | Separated/Widowed | 3 (4.8%) | 4 (2.5%) | 7 (3% |  |
| Type of leprosy | PB | 25 (40.3%) | 26 (16.5%) | 51 (23%) | 0.000 |
|  | MB | 37 (59.7%) | 132 (83.5%) | 169 (77%) |  |
| Detection delay | 1 year & below | 28 (45.2%) | 49 (31%) | 77 (35%) | 0.140 |
|  | 1 to 2 years | 25 (40.3%) | 79 (50%) | 104 (47%) |  |
|  | Above 2 years | 9 (14.5%) | 30 (19%) | 39 (18%) |  |
| WHO disability grade | Grade 0 | 43 (69.4%) | 88 (55.7%) | 131 (60%) | 0.032 |
|  | Grade I | 12 (19.4%) | 26 (16.5%) | 38 (17%) |  |
|  | Grade II | 7 (11.3%) | 44 (27.8%) | 51 (23%) |  |
| EHF score | 0 | 43 (69.4%) | 88 (55.7%) | 131 (59.5%) | 0.074 |
|  | 1-2 | 16 (25.8%) | 47 (29.7%) | 63 (29%) |  |
|  | 3 & above | 3 (4.8%) | 23 (14.6%) | 26 (12%) |  |
| Visible patch | Present | 56 (90.3%) | 112 (70.9%) | 168 (76%) | 0.002 |
|  | Absent | 6 (9.7%) | 46 (29.1%) | 52 (24%) |  |
| Ulcer | History of ulcer | 3 (4.8%) | 34 (21.5%) | 37 (17%) | 0.003 |
|  | No ulcers | 59 (95.2%) | 124 (78.5%) | 183 (83%) |  |

* Kuccha house– hut / thatched roof. ^Pucca house– house with concrete / tin sheeted / tiled roof.

Table 2: Difference between patients who were registered in government and TLM (for continuous variables)

| **Demographic and clinical characteristics*** | **Government**  **(n=62)** | **TLM**  **(n=158)** | **Total**  **(n=220)** | **t-test**  **(p-value)** |
| --- | --- | --- | --- | --- |
| Age | 38.2 (12.7) | 39.8 (14.1) | 39.4 (13.8) | 0.434 |
| Years of education | 7.9 (4.5) | 6.2 (4.9) | 6.7 (4.8) | 0.023 |
| Number of family members | 5.2 (2.1) | 4.6 (2) | 4.7 (2) | 0.041 |
| Monthly family income | 8445 (12044) | 4753 (5162) | 5794 (7891) | 0.002 |
| Detection delay in months | 17.3 (9.8) | 20.1 (14.5) | 19.3 (13.4) | 0.164 |
| EHF score | 0.65 (1.01) | 1.03 (1.3) | 0.92 (1.2) | 0.042 |
| PHQ-9 score | 8.95 (4.84) | 6.57 (5.15) | 7.24 (5.17) | 0.002 |
| GAD-7 score | 7.1 (4.17) | 5.83 (4.61) | 6.18 (4.52) | 0.064 |

*as mean (standard deviation)

Table 3: Association between duration of disability and presence of depression and anxiety (n=89).

|  | Depression | No depression | p-value | Anxiety | No anxiety | p-value |
| --- | --- | --- | --- | --- | --- | --- |
| Less than one year | 12 (57%) | 9 (43%) | 0.296 | 5 (24%) | 16 (76%) | 0.388 |
| One year or more | 30 (44%) | 38 (56%) |  | 23 (34%) | 45 (66%) |  |
|  | 42 (47%) | 47 (53%) |  | 28 (32%) | 61 (69%) |  |

Only those with disability either grade 1 or 2 were included in this analysis.
